# Ubiquitin-conjugating Enzymes in Cancer

**DOI:** 10.7150/ijbs.130297

**Published:** 2026-03-04

**Authors:** Zhiyang Yao, Ti Peng, Hao Dong, Yong Liao, Kai Miao, Jiang-Jiang Qin, Xiaoqing Guan

**Affiliations:** 1 College of Pharmaceutical Sciences, Zhejiang University of Technology, Hangzhou, Zhejiang, 310014, China.; 2 Center for Innovative Drug Research, Hangzhou Institute of Medicine (HIM), Chinese Academy of Sciences, Hangzhou, Zhejiang, 310018, China.; 3 School of Molecular Medicine, Hangzhou Institute for Advanced Study, UCAS, Hangzhou, Zhejiang, 310024, China.; 4 MOE Frontier Science Centre for Precision Oncology, University of Macau, Macau SAR, 999078, China.; 5 Department of Hepato-Pancreato-Biliary & Gastric Medical Oncology, Zhejiang Cancer Hospital, Hangzhou, Zhejiang, 310022, China.

**Keywords:** E2 ubiquitin-conjugating enzyme, cancer, target, mechanism.

## Abstract

Ubiquitin-conjugating enzymes (E2s) are emerging as critical regulators of oncogenic signaling networks by modulating protein stability, localization, and interactome dynamics. Distinct E2s orchestrate ubiquitin chain topology to drive cancer hallmarks including proliferation, immune evasion, metastasis, and metabolic reprogramming. Despite their central role in the ubiquitin-proteasome system, E2s remain underexploited as therapeutic targets. This review systematically maps E2-mediated signaling mechanisms across cancer types and proposes translational strategies for E2-targeted intervention.

## Introduction

Cancer remains a leading cause of global mortality, with >20 million new cases projected in 2025. Despite the success of kinase and immune-checkpoint inhibitors, most tumors eventually evade these agents through feedback reactivation of downstream signaling circuits 1-4, highlighting the urgent need to target alternative signaling layers that orchestrate oncogenic fate decisions. Targeting ubiquitination has emerged as an evolvable and druggable signaling stratum that writes, erases and re-wires oncogenic pulses.

**Figure 1:**
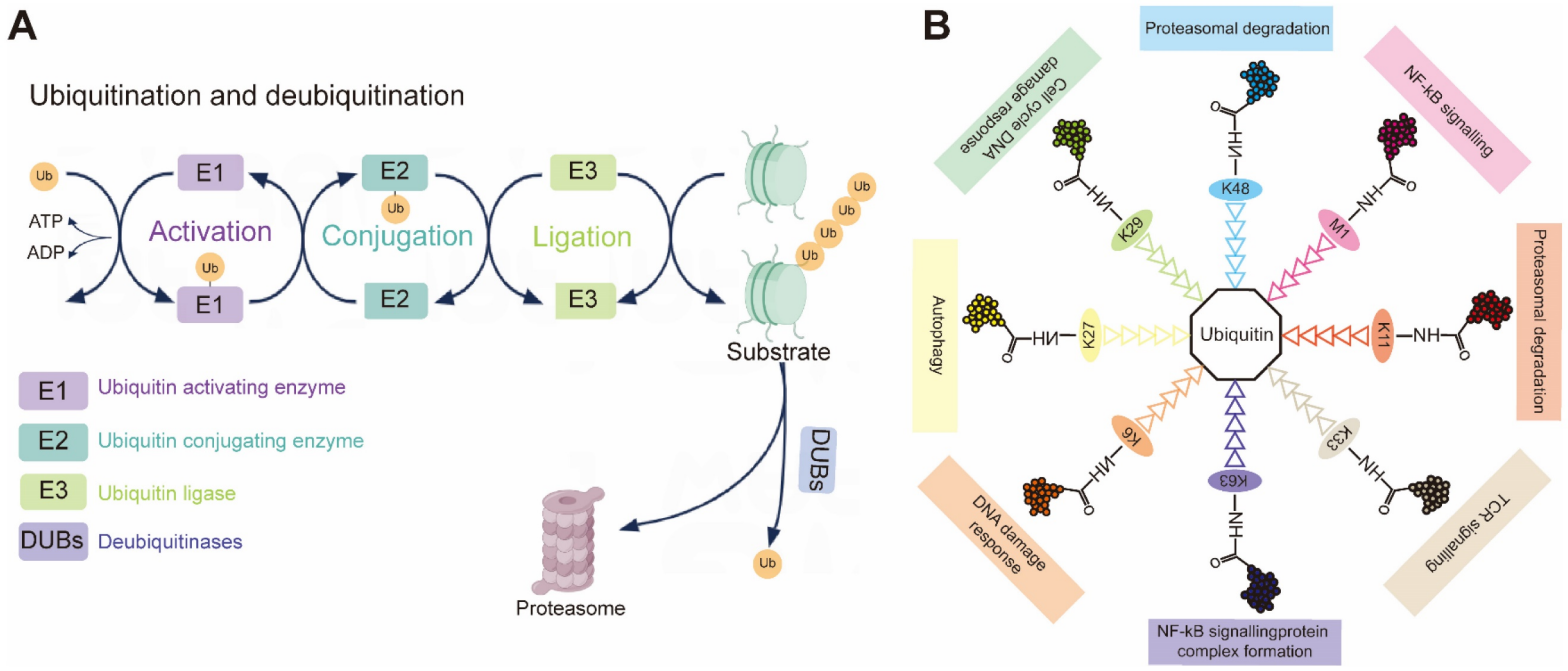
**(A)** Schematic demonstration of the ubiquitination process. Upon ATP-dependent activation, ubiquitin (Ub) is transferred from the E1 activating enzyme to the E2 conjugating enzyme. The E3 ubiquitin ligase then facilitates the final transfer and covalent attachment of ubiquitin to a specific substrate protein. The polyubiquitinated target is ultimately recognized and degraded by the 26S proteasome complex. **(B)** Individual ubiquitin linkage types. Ubiquitin a small 76-amino acid protein, can be attached to a targeted substrate or a ubiquitin molecule that is already attached to a substrate, resulting in specific polyubiquitin linkage types.

During ubiquitination, a cascade of E1 (activating), E2 (conjugating) and E3 (ligase) enzymes sequentially installs ubiquitin on substrates (Figure 1A). First, an E1-ubiquitin thioester bond is formed between the C-terminal Gly carboxyl group of ubiquitin and the active site Cys of the E1 through an ATP-dependent reaction. E1 then transfers the activated ubiquitin to the Cys residue of the E2 to form an E2-ubiquitin thioester linker intermediate by transesterification. Eventually, the E2-ubiquitin complex associates with an E3 ligase to deliver ubiquitin to a lysine, serine, threonine or even non-protein substrate 5, 6. In reality, the E2 step is a biochemical switchboard: each of the ~40 human members 7 selects which of eight possible chain linkages (K6, K11, K27, K29, K33, K48, K63 or M1) will be forged and how long or branched the resulting polymer will be (Figure 1B) 8, 9. Previous studies have shown that K11 and K48 chains lead to the degradation by the 26S proteasome, and that K63 chain is involved in various intracellular non-proteolytic functions, such as autophagy, DNA repair, and cell cycle control 10.

**Figure 2:**
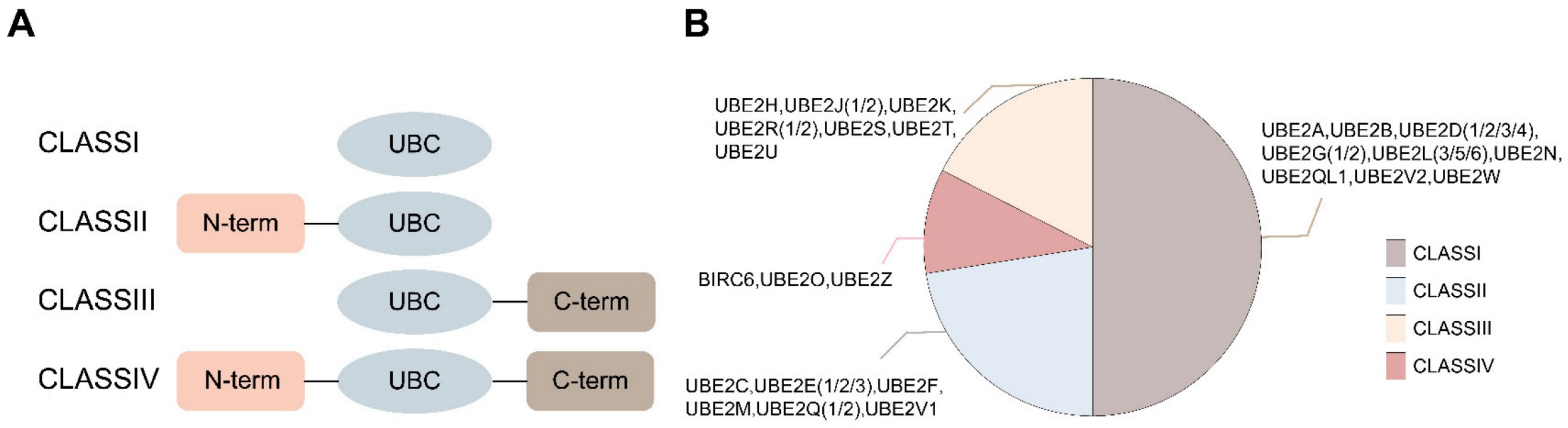
Classification of E2s. **(A)** Four classes of E2s are depicted in different framed boxes upon the absence (class I) or presence of additional extensions in the N- or C-terminal of the UBC domain (class II or class III, respectively) or in both termini (class IV). **(B)** All specific classifications of E2s.

E2s are structurally categorized into four classes: Class I (UBC catalytic core only), Class II (with an N-terminal extension), Class III (with a C-terminal extension), and Class IV (with both N- and C-terminal extensions) (Figure 2) 11. Most E2s can interact with E1 and one or more E3s, thereby providing a binding platform for E1, E3, and activated ubiquitin/ubiquitin-like (Ubl) proteins 12. Additionally, some E2s participate in non-canonical ubiquitin transfer processes, such as regulating the activity of other enzymes 13. Although all E2s share a conserved catalytic core composed of four antiparallel β-strands flanked by four α-helices, this structure can mediate diverse biological functions, including substrate degradation, scaffolding complex assembly, and even the formation of degradation-resistant ubiquitin chains that regulate kinase clustering, DNA repair foci assembly, or NF-κB signalosome activation.

The functional diversity of E2s is largely determined by their extension regions beyond the conserved catalytic core domain. These extensions serve as key structural determinants influencing E2 subcellular localization, the stability of their interactions with E1, and the regulation of E3 ligase activity. Taking the N-terminal extension as an example, it enables fine-tuning of E2 interactions with both E1 and E3. The N-terminal region of UBE2M enhances its binding to the NEDD8-specific E1 while reducing its affinity for the ubiquitin-specific E1 14. Similarly, the N-terminal extensions of E2s like UBE2C also modulate ubiquitin transfer efficiency by decreasing their affinity for E1 15. This structural feature may act cooperatively with the E2-E3 interaction interface to facilitate the ubiquitination of substrates lacking canonical degradation signals. Research indicates that deletion of the N-terminus in UBE2C leads to excessive ubiquitin transfer mediated by its E3 ligase APC/C, resulting in aberrant substrate degradation 15. Furthermore, the N-terminal region directly participates in E3 ligase recognition. For instance, UBE2L3 utilizes Arg5 and Arg15 within its N-terminal α1-helix to bind the E3 ligase CBL, thereby negatively regulating receptor tyrosine kinase signaling 16. The interaction between UBE2D2 and the E3 ligase CNOT4 depends on Lys4 within its N-terminal α1-helix 17.

On the other hand, C-terminal extensions are primarily involved in regulating enzymatic activity and ubiquitin chain elongation. For example, the C-terminal extension of UBE2R1 is essential for polyubiquitin chain formation, with recent studies suggesting its mechanism may involve interactions between C-terminal residues and the conjugated ubiquitin molecule 19. The acidic C-terminal tail of UBE2R2 can be phosphorylated by protein kinase CK2. This modification can affect SCF complex-mediated Sic1 ubiquitination, cell cycle progression, and potentially the intracellular distribution of this E2 20. Extension regions also play a crucial role in subcellular localization. Although UBE2G2 itself lacks a transmembrane domain, its C-terminal extension can specifically recognize the endoplasmic reticulum (ER) membrane protein Cue1. This interaction "anchors" UBE2G2 to the ER membrane, enabling efficient collaboration with ER-associated E3 ligases 21. Similarly, phosphorylation of the C-terminal tail of UBE2R2 is also involved in regulating its intracellular localization 22.

E2s are not only key pathogenic factors in neurodegenerative diseases 11, chromosomal instability syndromes 23, and autoimmune disorders 24, but also are implicated in every cancer hallmark, promoting cancer initiation and progression. E2 dysregulation has been documented in a wide spectrum of cancers. Moreover, numerous E2s drive cell cycle progression, DNA repair, and stimulate oncogenic signaling pathways during malignant transformation. Consequently, E2s are increasingly recognized as valuable cancer-related biomarkers for diagnosis, prognosis, and therapeutic targeting 25.

Here, we synthesize current knowledge on how E2s sculpt the linkage-specific transduction networks that drive each hallmark of cancer. We then inventory existing small-molecule inhibitors and miRNA mimics/antagomirs that directly engage E2s or their 3'-UTRs, summarizing their selectivity and *in vitro* and *in vivo* efficacy. Finally, we chart emerging research directions that will shape the next decade of E2-centric clinical development.

### E2 mutations in cancer

Gene mutations 26, 27 are one of the fundamental drivers of biological phenotypic diversity and disease onset. Mutations are broadly categorized into germline mutations and somatic mutations 28: Germline mutations are heritable and constitute the root cause of numerous genetic disorders; whereas somatic mutations, which occur in somatic cells, are a key driver in tumorigenesis and progression. In cancer research, analyzing mutation profiles in specific genes—such as proto-oncogenes and tumor suppressor genes—not only reveals the molecular mechanisms of tumorigenesis but also provides critical theoretical foundations for targeted therapies and biomarker development. The subject of this paper, E2s exhibit relatively rare mutations. However, these mutations play pivotal roles in the onset and progression of various cancers. Different types of E2 mutations disrupt normal catalytic function, substrate recognition, or protein interactions, leading to specific pathological processes.

*UBE2A*: Somatic mutations in the *UBE2A* gene at two nonadjacent residues (D114V and I33M) have been identified in CML. These mutations cause conformational changes in the UBE2A protein, resulting in the loss of its ability to regulate the activity of downstream target proteins. Compared to the WT, the mutant *UBE2A* exhibits significantly reduced ATP consumption and correspondingly weaker ubiquitin-binding activity, leading to a loss of regulatory control over CSF3R transcription. CSF3R, a key regulator of myeloid lineage differentiation and development 29, is affected by UBE2A dysfunction, which may hinder normal cell differentiation by interfering with granulocyte development-related pathways 30. Notably, *UBE2A* mutations are not exclusive to CML. They have also been observed in patients with DLBCL 31. In Chinese DLBCL cases, *UBE2A* mutations account for approximately 10% of instances and are found exclusively in patients with bone marrow involvement 32. Furthermore, these mutations are significantly associated with a shortened time to transformation into an aggressive FL subtype following biopsy confirmation 33. Additionally, *UBE2A* has been reported as a potential novel driver gene in cervical cancer 34. This cross-cancer evidence suggests that interventions targeting UBE2A may represent a potential auxiliary therapeutic strategy, although the specific mechanisms underlying its oncogenic role require further elucidation.

*UBE2C*: UBE2C is widely overexpressed in multiple cancer cell lines and tissues, and its elevated expression is significantly associated with tumor proliferation, invasion, metastasis, and poor clinical prognosis 35. Mutations in this gene are commonly observed in various solid tumors, including CRC, EC, GC, BCa, NSCLC, SARC, MEL, PC, and GBM. Pan-cancer analysis based on the cBioPortal database reveals diverse forms of genetic alterations in* UBE2C*, primarily including gene amplification, point mutations, deep deletions, and multiple alterations, with gene amplification being the most predominant variant type 36. These genetic alterations, particularly mutations and amplifications, are considered key factors driving the abnormally high expression of UBE2C in tumors such as UCEC. In PRAD, CNVs of *UBE2C* are significantly associated with patients' progression-free survival (PFS), disease-specific survival (DSS), and disease-free survival (DFS), indicating its important prognostic value. Furthermore, abnormal mRNA expression of *UBE2C* is mainly associated with missense mutations and splicing mutations, which are predominantly distributed in diploid regions and copy number gain regions of the genome 36.

*UBE2G2*: Menezes et al. identified deleterious mutations of *UBE2G2* in human leukemia for the first time 37. Targeted resequencing results revealed a single-amino-acid-change mutation in the *UBE2G2* gene, specifically an allele T>A variation, with a mutation frequency of 4% in the study cohort. The impact of this mutation on downstream signaling pathways requires further investigation.

*UBE2I*: UBE2I regulates the proliferation and transformation of different cancers and is targeted by a variety of viruses, including HIV, EBV, and HPV 38, 39. A large number of novel missense mutations in *UBE2I* have been identified in cancer patients, but their functional implications are unclear 40, 41.

*UBE2M*: SNVs or CNVs in the *UBE2M* gene can be observed across multiple cancer types 42. Particularly in LUSC, SNVs in *UBE2M* correlate with lower patient survival rates, while its CNVs also indicate poorer clinical prognosis. Mutated UBE2M modulates neddylation pathways by regulating NEDD8 binding, thereby contributing to tumorigenesis and progression. Functionally, UBE2M primarily promotes cell cycle progression while inhibiting RAS/MAPK and RTK signaling pathways.

*UBE2O*: Mutations in *UBE2O* are relatively common across multiple cancers, including breast, gastric, renal, and ovarian cancers 43. Analysis of data from The Cancer Genome Atlas (TCGA) database indicates that *UBE2O* gene amplification frequently occurs in various cancer types, such as gastric and lung cancers. This amplification drives cancer cell proliferation by promoting the ubiquitination and degradation of AMPKα2 44. *UBE2O* mutations, including potential splicing variants and other genetic defects, may impact its biological functions, such as regulation of cell proliferation and apoptosis. However, the role of *UBE2O* mutations in cancer initiation and progression remains to be fully explored.

*UBE2S*: Huijie Bian 45 et al. identified two novel somatic mutations in *UBE2S* (p.Gly57Ala and p.Lys63Asn) via whole-exome sequencing. Although researchers have not yet investigated the molecular mechanisms of these mutant *UBE2S* variants in depth, scale-invariant feature transformation analysis predicted functional effects, suggesting that these two amino acid substitutions may have adverse consequences.

*UBE2T*: Analysis of large-scale genomic and expression profiling data covering hundreds of cancer cell lines in the cBioPortal platform reveals that the *UBE2T* gene is altered in multiple cancers, with particularly prominent high-frequency amplification 46. Taking breast cancer as an example, UBE2T exhibits significant overexpression at both the transcriptional and protein levels 47. Its potential oncogenic mechanism may involve polyubiquitination modification of the E3 ligase RING ligase BRCA1—a key tumor suppressor gene in breast cancer development—and subsequent promotion of its degradation via the proteasome pathway. Furthermore, a novel frameshift mutation c.415_418insAGCC in *UBE2T* was identified in germ cells from breast/ovarian cancer patients 48. This mutation introduces 22 additional amino acids downstream, resulting in loss-of-function protein.

*UBE2W*: Yan Yuan et al. 49 found that 20% of BC patients exhibited alterations in the *UBE2W* gene, with significant differences in mRNA expression levels across all analyzed samples. Further analysis revealed markedly elevated mRNA expression in cases with *UBE2W* gene amplification. Additionally, patients harboring *UBE2W* mutations exhibited significantly shorter overall survival and recurrence-free survival compared to those without this genetic alteration. These findings suggest that UBE2W may influence clinical outcomes in BC patients by interacting with the E3 ligase RBX1.

The pathogenic significance of E2 mutations extends far beyond their low frequency in pan-cancer genomic landscapes. While these mutations occur sparsely, they can function as potent drivers of oncogenesis through diverse mechanisms—including catalytic dysregulation, dosage-dependent effects, and altered protein interaction interfaces. Consequently, E2 mutations may serve as critical determinants of tumor progression, therapy resistance, and immune evasion in specific contexts. However, the direct role of these mutations in cancer progression has not been fully explored.

Even mutations that appear infrequently in pan-cancer genomic maps may carry significant pathogenic implications. For instance, IDH1/2 mutations occur as low-frequency missense mutations in gliomas and acute myeloid leukemia, yet their mutation sites are located at the enzyme's active site, directly impacting its catalytic properties 50. IDH1/2 mutations reduce α-ketoglutarate production capacity and confer an abnormal function—converting α-ketoglutarate into (R)-2-hydroxyglutarate ((R)-2HG) using NADPH as a cofactor 51, 52. (R)-2HG is considered a primary oncogenic metabolite, exerting multiple biological effects in IDH1/2-mutant tumors. Consequently, IDH1/2 mutations are regarded as gain-of-function alterations 51. Similarly, under specific contexts, E2 mutations may serve as critical determinants of tumor progression, therapeutic resistance, and immune evasion. However, the mechanisms underlying the role of these E2 mutations in cancer progression remain poorly understood.

## E2s in the hallmarks of cancer

The occurrence and development of cancer is a complex process that involves a variety of factors and mechanisms. The publication of the study 159 by Douglas Hanahan and Robert A. Weinberg et al. describing the top ten hallmarks of tumor cells has become a hot topic in oncology. The role of E2 has also become increasingly important in the study of the top ten features of cancer. Although the relationships between cancer hallmarks are complex and often interdependent, we nonetheless attempt to decipher the E2s associated with each specific hallmark. This approach aims to provide illustrative examples for each marker, thereby advancing our understanding of cancer pathogenesis and informing potential therapeutic strategies (Table 1).

### E2s in sustained proliferation signaling

#### E2s and cell cycle regulators

The cell cycle is governed by a series of checkpoints—namely, the G1/S, G2/M, and spindle assembly checkpoint (SAC)—which collectively ensure orderly progression from one phase to the next 160. Dysregulation of these checkpoints is critically implicated in the initiation and progression of cancer. Importantly, the cell cycle is extensively modulated by the UPP.

**Figure 3:**
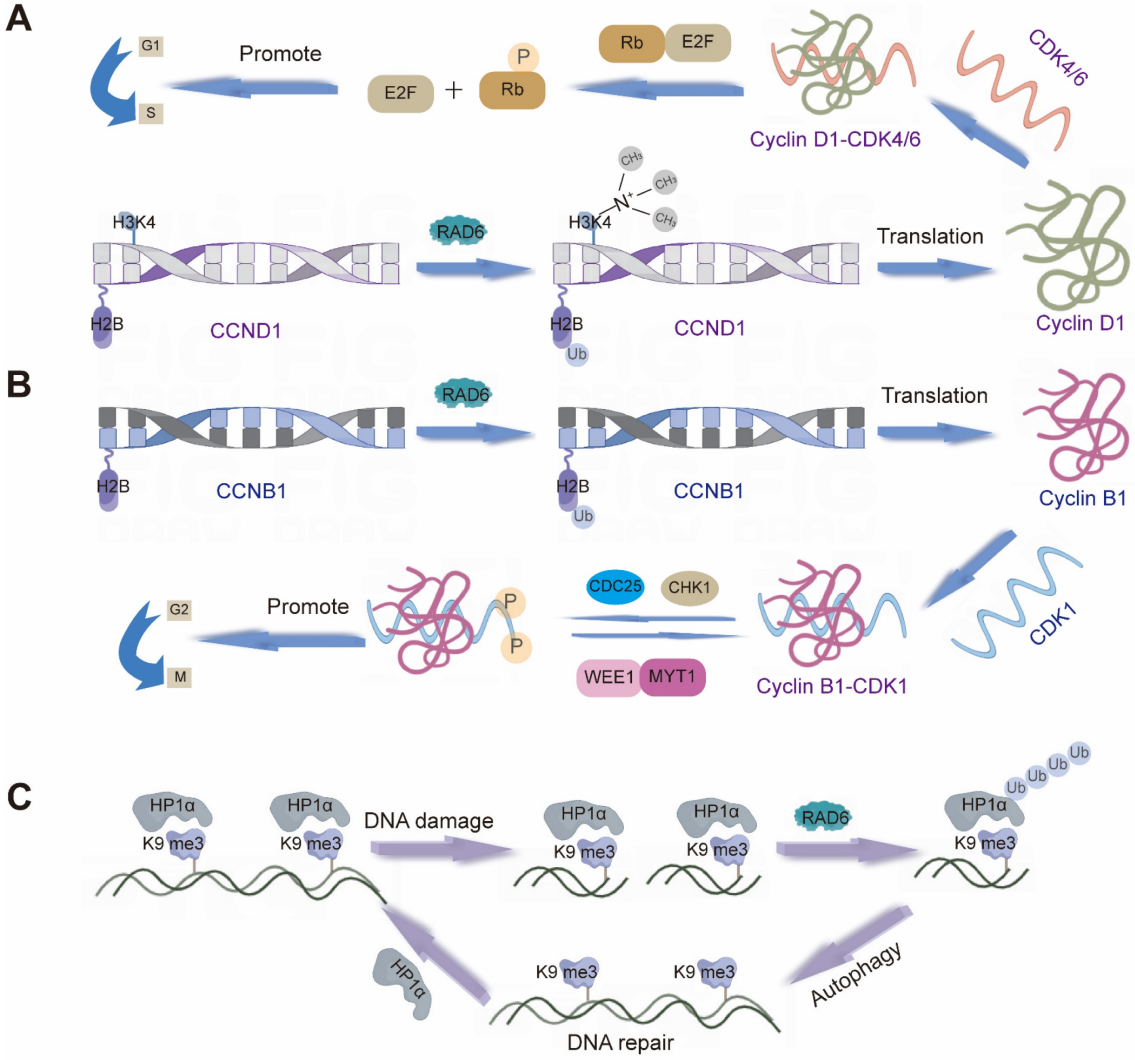
Different mechanisms of action of RAD6. **(A)** RAD6 regulates the transcription of *CCND1* likely through affecting the H2B monoubiquitination and H3K4me3 levels at the *CCND1* promoter region. **(B)** RAD6 regulates the transcription of *CCNB1* likely through affecting the H2B monoubiquitination. **(C)** RAD6 interacts physically with HP1α, ubiquitinates HP1α at K154, and further degrades HP1α through the autophagy-lysosome pathway.

Cyclin B1 serves as a pivotal regulator throughout the cell cycle. It forms a complex with CDK1 to drive mitotic entry. During early M phase, the Cyclin B1/CDK1 complex triggers nuclear envelope breakdown and chromosome condensation, thereby promoting mitosis 161, 162. Several E2s are intimately linked to Cyclin B1 expression and function, influencing both cell cycle control and tumorigenesis. For example, UBE2A upregulates *CCNB1* (which encodes Cyclin B1) via H2B monoubiquitination, modulating Cyclin B1 protein levels (Figure 3B) 163. Similarly, UBE2C expression shows a strong positive correlation with Cyclin B1. Downregulation of UBE2C reduces cyclin B1 and CDK1 levels by blocking the ERK/AKT signaling pathway, thereby diminishing MPF complex formation and arresting the cell cycle at the G2/M phase. This also inhibits tumorigenesis 164. In hepatocellular carcinoma, UBE2T exhibits a mechanism similar to that of UBE2C described above. Furthermore, studies have revealed that inhibiting UBE2T leads to downregulation of p21 and p27—both members of the Cip/Kip family of cyclin-dependent kinase inhibitors 165.

Cyclin D1 is another essential cell cycle regulator, primarily active during the G1 phase. It forms complexes with CDK4 and CDK6, known collectively as Cyclin D1-CDK4/6 complexes 166. These complexes phosphorylate the retinoblastoma protein (Rb) and other downstream targets to drive G1 progression 167. Multiple E2s, including UBE2A, UBE2D3, and UBE2M, contribute to the regulation of Cyclin D1. UBE2A, for example, enhances H2B ubiquitination and H3K4me3 levels at the *CCND1* promoter, leading to its transcriptional upregulation (Figure 3A) 168. UBE2D3 (UBE2D3-hTERT-cyclin D1) and UBE2M (Wnt/β-catenin pathway) are believed to influence cyclin D1 stability through distinct mechanisms, thereby participating in cell cycle control 169, 170. Together, these enzymes fine-tune Cyclin D1 activity and play coordinated roles in cell proliferation. Beyond positive regulators such as Cyclins B1 and D1, negative cell cycle regulators also play crucial roles in maintaining cellular homeostasis by restraining proliferation. E2s are similarly involved in modulating these inhibitory factors. For example, Reports indicate that UBE2D1 mediates the ubiquitination and degradation of the tumor suppressor protein p53 both *in vitro* and *in vivo*. *In vitro*, it interacts with MDM2 to trigger the ubiquitination process of p53. UBE2D1 participates in the ubiquitination of HSP90AB1, which contributes to the stability of p53 and its transport to the cell nucleus 171. P27^kip1^ is a member of the Cip/Kip family of CKIs. UBE2I can cooperate with CRM1 to mediate SUMOylation of p27^kip1^, thereby regulating its nuclear export and influencing cell cycle progression 99. Moreover, knocking down UBE2M inhibits neddylation modification of Cullin proteins, leading to inactivation of CRL E3s and inducing accumulation of tumor-suppressive CRL substrates (p21, p27, and Wee1). This induces cell cycle arrest and suppresses the malignant phenotype of lung cancer cells 172.

#### E2s and signaling pathways

Cell growth signaling pathways play crucial roles in maintaining life processes such as cell proliferation, differentiation and survival. Their aberrant activation or inhibition is usually closely associated with the occurrence and development of various diseases, especially cancer 173. Wnt/β-catenin and NF-κB are some common cellular growth signaling pathways. E2s are directly or indirectly involved in cell proliferation, growth and cancer development by regulating the activity of these signaling pathways.

The Wnt/β-catenin signaling pathway constitutes a fundamental signaling cascade that is essential for numerous physiological processes, including embryonic development, tissue regeneration, and cellular homeostasis 174. Dysregulation of this pathway, due to genetic or epigenetic alterations, is a well-established driver in various human cancers, such as colorectal, breast, and gastric cancer. A pivotal event in pathway activation is the nuclear accumulation of β-catenin, which results from the functional impairment of the β-catenin destruction complex. E2s are critical regulators of the Wnt/β-catenin pathway. For instance, UBE2S acts as a novel pathway activator by catalyzing a Lys11-linked polyubiquitin chain on β-catenin at Lys19. This specific modification stabilizes β-catenin by antagonizing its Lys48-linked ubiquitination and subsequent proteasomal degradation, which is normally mediated by the destruction complex and β-TrCP 175. The aberrant activation of this pathway, primarily driven by the nuclear translocation of β-catenin, is a hallmark of many tumors, where it promotes oncogenic gene transcription, cell proliferation, and stem cell self-renewal 162, 176. The pathological significance of E2s is further underscored by their roles in different cancer contexts: UBE2A and UBE2B influence the pathogenesis of melanoma and breast cancer by modulating the Wnt/β-catenin pathway 177, while UBE2M and UBE2S contribute to its activation in malignancies such as ovarian cancer 178, 179.

**Figure 4:**
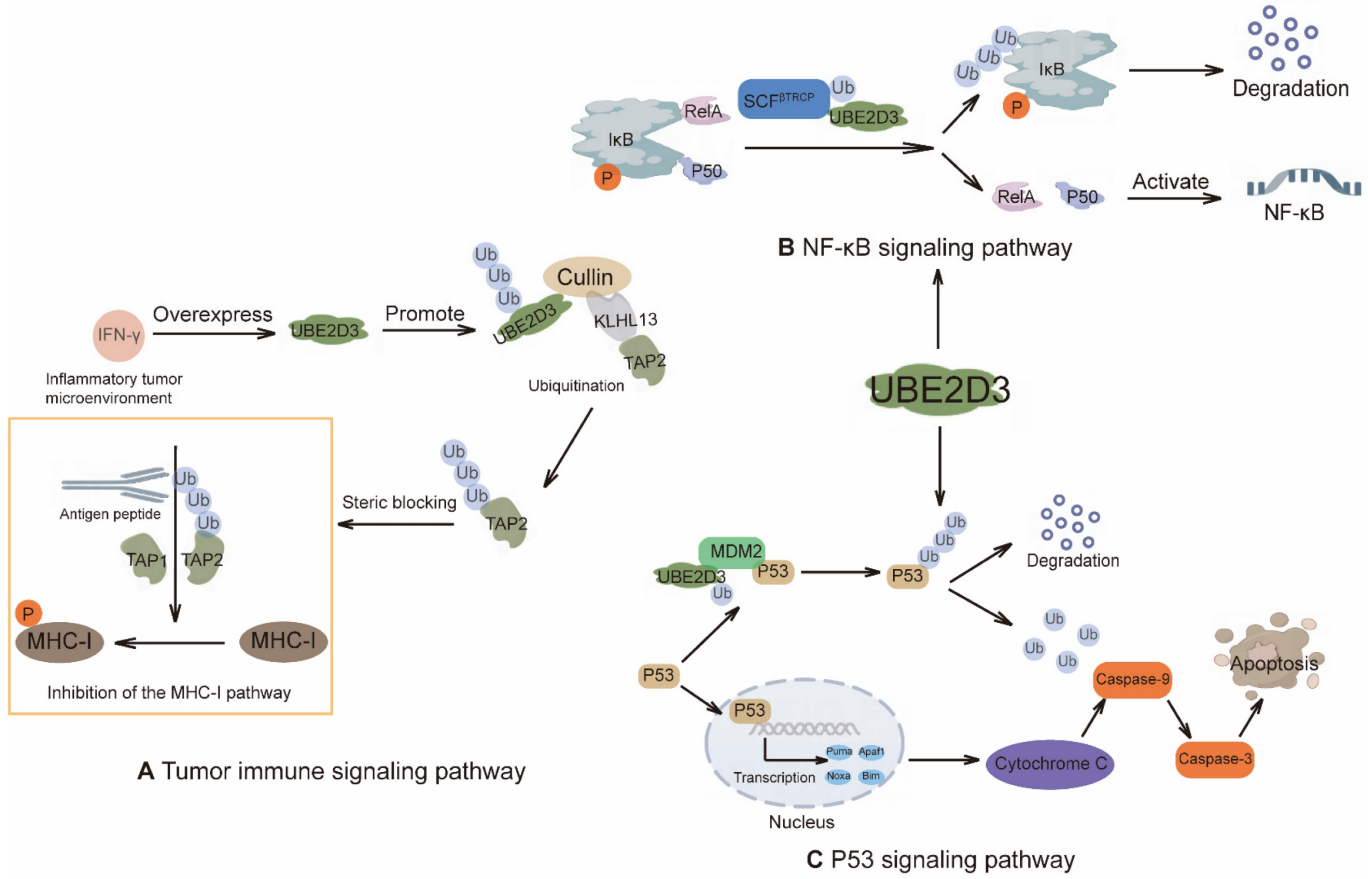
Different mechanisms of action of UBE2D3. **(A)** IFN-γ-driven UBE2D3 upregulation impairs antigen presentation pathways and antitumor immunity in pancreatic cancer. **(B)** NF-κB/IκB pathway: IκB forms an inhibitory complex with the NF-κB subunits RelA and p50. Phosphorylation of IκB promotes its ubiquitination by the E3 RNF138 in conjunction with UBE2D3, leading to proteasomal degradation of IkB and activation of NF-κB target genes. **(C)** The role of UBE2D3 in the p53 signaling pathway. The p53 tumor suppressor regulates gene expression and ultimately induces apoptosis.

The NF-κB family comprises transcription factors that play critical roles in a range of biological processes, including inflammation, immune response, and apoptosis 180. Beyond its canonical signaling through NF-κB, this system also exerts effects via interferon regulatory factors 181. Numerous cellular signaling pathways converge on NF-κB, and ubiquitination plays a crucial role in coordinating these signals to regulate NF-κB activity. In this pathway, the UBE2N-UBE2V1 complex synergistically mediates K63-linked polyubiquitin chain formation with CHIP, enhancing its activity in promoting TACE maturation and thereby blocking TNFα-induced NF-κB signaling 182. Dysregulation of the NF-κB pathway has been strongly implicated in the development and progression of multiple cancers 183. E2s such as UBE2D1 and UBE2D3 (Figure 4B) contribute to the precise modulation of this pathway, influencing cancer cell proliferation, survival, and metastatic behavior 184, 185. Given its central role in oncogenesis, the NF-κB pathway—and its regulatory E2s—represents a promising target for therapeutic intervention in cancer.

### Role of E2s in replication immortality

One of the characteristics of cancer cells is their abnormal cell proliferation and replication capacity 186. Normal cells have a limited ability to proliferate under certain conditions, whereas cancer cells can proliferate and divide without restriction to form tumor tissue. Replicative immortality of cancer cells is associated with telomerase activity 187. Telomerase is an enzymatic protein that protects telomeres at the ends of chromosomes. In normal cells, telomeres are progressively shortened each time the cell divides due to limitations in DNA replication, which eventually leads to cellular senescence and death. However, in cancer cells, telomerase activity is abnormally increased and telomeres are maintained at a constant length, allowing the cancer cells to divide indefinitely and achieve "replicative immortality" 188. This aberrant expression of telomerase activity is considered to be an important feature of cancer cells and one of the key mechanisms by which cancer cells are able to sustain growth and spread. Therefore, the regulation of telomerase activity has become one of the important research directions in cancer therapy.

In telomere maintenance, ubiquitination modulates telomere length and stability by regulating the stability and function of telomere-associated proteins 189. In the regulation of telomeric chromatin by ubiquitin, post-translational modifications of histone tails—such as methylation and acetylation—serve as potent mechanisms governing chromatin structure. It has been discovered that RAD6 regulates H3K4-me by adding a ubiquitin molecule to lysine 123 of histone H2B (monoubiquitination of H2B) 190. As the catalytic subunit of telomerase, the core function of hTERT is to maintain telomere length directly by adding telomeric repeats to chromosome ends in a reverse transcription manner 191. Studies have reported that UBE2D3 can stimulate hTERT degradation via ubiquitin-dependent proteolysis, leading to reduced telomerase activity and ultimately resulting in telomere shortening 85. Although the maintenance of telomere length is closely related to ubiquitination, the role of E2s in the regulation of telomerase remains to be further explored.

### Role of E2s in evading growth inhibition

Cancer cell evasion of growth inhibition refers to the process whereby tumor cells escape normal regulatory mechanisms and ignore inhibitory signals, resulting in uncontrolled proliferation and dissemination 192. This phenomenon plays a crucial role at different stages of tumor development, especially during early tumor formation and progression. The mechanism by which cancer cells escape growth inhibition involves multiple aspects, including cell cycle regulation, DNA repair, apoptosis and other biological processes 193. Cancer cells can evade growth inhibition by regulating the expression of relevant genes, post-translational modification of proteins and activation of signal transduction pathways. These aberrant growth regulatory mechanisms enable cancer cells to continue to proliferate and spread unchecked by external factors, eventually forming malignant tumors 194.

Research indicates that multiple E2s participate in tumor cell escape from growth inhibition by regulating the ubiquitination of key tumor suppressor proteins such as p53 and p27. The stability of p53 is precisely regulated by ubiquitination, and its abnormal degradation is closely associated with tumorigenesis. For instance, significant overexpression of the UBE2T has been observed in various cancers including HCC, CRC, and GC. UBE2T catalyzes K48-or K63-linked ubiquitination of p53, thereby promoting its degradation or altering its function. This ultimately disrupts p53-dependent downstream signaling pathways, weakening its growth-inhibitory and pro-apoptotic effects 116. Conversely, the ubiquitin-mediated degradation of the cyclin-dependent kinase inhibitor p27 is a critical step in releasing cell cycle arrest and driving cells into the DNA replication phase. Research indicates that UBE2S interacts with the scaffold protein TRIM28 within the nucleus, synergistically enhancing p27's ubiquitination levels and accelerating its degradation 45. The loss of p27 removes inhibition on the cyclin-kinase complex, propelling cell cycle progression and enabling cells to bypass normal proliferation surveillance.

The abnormally high expression of these E2s disrupts the homeostasis of core tumor suppressor proteins like p53 and p27, thereby undermining critical biological processes such as cell cycle checkpoints, DNA damage response, and apoptosis pathways. This creates favorable conditions for the uncontrolled proliferation and dissemination of tumor cells.

### Role of E2s in the regulation of cell death

The UPP plays a crucial role in the regulation of apoptosis, a process essential for maintaining cellular homeostasis under various dysregulated conditions 184. Key apoptotic regulators include the BCL-2 protein family, IAPs, and the IKK complex 195. Apoptosis primarily proceeds through two major pathways: the extrinsic and the intrinsic routes. The extrinsic pathway is initiated by extracellular signals such as death ligands (TNF, FASL), cytokines, cytotoxic stimuli, or alterations in the extracellular matrix 196. These signals are transduced via cell surface receptors, leading to caspase activation and the execution of apoptosis 197. In contrast, the intrinsic pathway is triggered by intracellular stressors, including DNA damage, metabolic dysfunction, or cell cycle dysregulation. These stimuli induce mitochondrial outer membrane permeabilization, resulting in the release of pro-apoptotic factors such as cytochrome C and BCL-2 family proteins.

In MM, the transcription factor c-Maf is frequently overexpressed in more than 50% of cell lines and patient samples, primarily due to chromosomal translocations and other mechanisms 198. Notably, UBE2O has been identified as a key regulator that induces K48-linked polyubiquitination of c-Maf, suppresses its transcriptional activity, and thereby promotes MM cell apoptosis while inhibiting tumor growth 199. As a central tumor suppressor, p53 regulates apoptosis through the transcriptional control of genes such as PUMA, NOXA, BIM, BAX, BCL2, FAS, and IGFBP3, and can also trigger apoptosis via death receptor pathways like those involving TNF and Fas 200. For instance, UBE2D3 cooperates with MDM2 to conjugate K48-linked polyubiquitin chains onto p53, triggering its proteasomal degradation. This modulates the diverse activities of p53, including its ability to promote apoptosis 201. Furthermore, inhibiting UBE2J1 can regulate the PI3K/AKT and MDM2/p53 signaling pathways to promote apoptosis in endometrial cancer 202.

BAX, a key apoptotic effector, undergoes conformational activation and mitochondrial translocation upon cellular stress, forming pores that facilitate cytochrome c release and apoptosome formation 203. UBE2L3 promotes the ubiquitin-mediated proteasomal degradation of GSK3β, the loss of GSK3β blocks p65 activation. This leads to the upregulation of p65 target proteins such as Bax, ultimately influencing apoptosis in HCC 124. Similarly, the pro-apoptotic protein NOXA, which enhances cellular sensitivity to apoptosis, is regulated by UBE2F-mediated ubiquitination and degradation. UBE2F pairs with SAG/RBX2 to mediate neddylation of CUL5, thereby activating the CRL5 E3 complex. Activated CRL5 then conjugates K11-linked ubiquitin chains to NOXA, targeting it for degradation and consequently inhibiting apoptosis to enhance cell survival 204. Unlike other BCL-2 family members, NOXA expression is subject to multi-level regulation, including transcriptional control, post-translational modification, and protein degradation 205. Its overexpression promotes apoptosis in numerous cancer types, highlighting its potential as a therapeutic target in oncology 206.

### Role of E2s in immune escape

Immune escape is a key process in tumor development that involves tumor cells using various mechanisms to avoid detection and clearance by the immune system, thereby promoting tumor growth, metastasis and treatment resistance 207. Multiple E2s, including UBE2D3, UBE2I, UBE2N, UBE2S, UBE2L6 and UBE2F are implicated in this process. These E2s may influence cells' ability to evade the immune system by regulating antigen presentation, immune checkpoint/co-stimulatory molecules, cytokine/chemokine signaling pathways, and the shaping of an immunosuppressive microenvironment.

Our recent studies have identified UBE2D3 as a key driver of inflammation-dependent immune escape in pancreatic cancer. The underlying mechanism involves the inflammatory microenvironment inducing UBE2D3 overexpression in tumor cells, which then associates with the E3 ligase KLHL13 to mediate K63-linked polyubiquitination of lysine 245 on the transporter associated with antigen processing 2 (TAP2). This modification creates steric hindrance that impairs the peptide-transport function of TAP2, thereby blocking the antigen presentation pathway and facilitating immune evasion by tumor cells 141 (Figure 4A).

Immune checkpoints refer to a class of immune inhibitory molecules expressed on immune cells, which fine-tune the degree of immune activation and prevent the occurrence of autoimmunity. The expression of immune checkpoint molecules is precisely regulated by E2s. In prostate cancer, UBE2I within macrophages mediates SUMOylation modification at lysine 350 of STAT4, promoting its ubiquitination and degradation. This subsequently inhibits macrophage polarization towards the M1 phenotype via the JAK/STAT signaling pathway. Application of the UBE2I inhibitor 2-D08 enhances the anti-tumor function of tumor-associated macrophages, stimulates the proliferation and activation of CD8⁺ T cells, and upregulates PD-1 expression, demonstrating synergistic potential when combined with immune checkpoint blockade therapy 142. In liver cancer, miR-122-3p downregulates PD-L1 expression and enhances anti-tumor immune responses by targeting UBE2I and inhibiting the SUMOylation modification of the NF-κB signaling pathway 303. Conversely, inhibition of UBE2N in macrophages reduces K63-linked polyubiquitination, leading to AKT activation and upregulation of PD-L1 expression, thereby promoting tumor progression 144.

Beyond immune checkpoint blockade, targeting cytokine/chemokine signaling pathways represents another promising strategy in cancer immunotherapy. The IL-15 signal is crucial for maintaining the function of cytotoxic lymphocytes. UBE2F inhibits NK cell function and suppresses the secretion of pro-inflammatory cytokines by mediating the Neddylation modification of CUL5, which activates the CRL5 complex. This activated complex subsequently targets the IL-15 receptor for proteasomal degradation 113. IFN-I is also one of the most critical categories of cytokines in the immune system, playing a central role in initiating and coordinating early antiviral innate immune responses. Within the IFN-I pathway, UBE2S negatively regulates this signaling axis by recruiting USP15 to remove the K63-linked polyubiquitin chains from TBK1 208. Furthermore, RSK1-mediated phosphorylation of UBE2L6 alters its substrate preference, enabling it, in concert with RNF19A, to drive cGAS ubiquitination. This process consequently inhibits cGAS-STING pathway-mediated IFN-I production and intrinsic anti-tumor immunity 209.

In shaping the immunosuppressive tumor microenvironment, UBE2S also plays a significant role. The expression level of UBE2S shows a positive correlation with the infiltration of M2-polarized tumor-associated macrophages, suggesting its potential as a biomarker for predicting responses to immunotherapy 210. Mechanistically, research has revealed that UBE2S recruits USP10 through the N-terminal domain of USP10. Together, they regulate the K48-linked deubiquitination of the GLUT1 protein, thereby stabilizing GLUT1 and enhancing glycolytic activity. This metabolic reprogramming leads to lactate accumulation, which in turn induces the differentiation of M2-polarized macrophages and the secretion of TGF-β1. These changes further promote the transformation of fibroblasts into myofibroblasts, accelerating the process of tissue fibrosis 211.

Although current research on the specific mechanisms by which E2s affect immune escape remains insufficient, these E2s likely play a significant role in the immune escape process of tumor cells by regulating the expression of immune-related genes, influencing immune-related signaling pathways, and promoting tumor growth and metastasis.

### Role of E2s in angiogenesis

Normally, angiogenesis is a temporary process that occurs only under specific physiological and pathological conditions, such as wound healing and endometrial proliferation. However, cancer cells can manipulate and promote angiogenesis through different mechanisms to meet their nutritional needs for continued growth and spread 212. In angiogenesis, E2s play a key role.

Vascular endothelial growth factor receptor 2 (VEGFR2) serves as a central regulator of endothelial cell function and angiogenesis. During angiogenesis, the E2 ubiquitin-conjugating enzymes UBE2D1 and UBE2D2 modulate VEGFR2 ubiquitination and fine-tune endothelial responses to VEGF-A signaling. Inhibition of either UBE2D1 or UBE2D2 elevates the steady-state level of VEGFR2, thereby potentiating VEGF-A-stimulated signal transduction and amplifying the activation of downstream pathways, including the classical MAPK cascade, phospholipase Cγ1, and the AKT pathway 81.

Beyond VEGFR2 regulation, UBE2D1 also influences angiogenesis—particularly in muscular contexts—by targeting hypoxia-inducible factor-1α (HIF-1α), a master cellular oxygen sensor and key transcriptional regulator of angiogenesis. Under normoxic conditions, HIF-1α undergoes proline hydroxylation at residues 402 and/or 564 catalyzed by prolyl hydroxylase domain proteins (PHDs) 1-4. This modification promotes its recognition by the Von Hippel-Lindau tumor suppressor (VHL) and subsequent ubiquitination facilitated by UBE2D1, leading to proteasomal degradation and maintenance of low basal levels. In contrast, under hypoxic conditions, PHD and VHL activities are suppressed, resulting in HIF-1α stabilization, transcriptional activation, and the promotion of angiogenesis, glycolysis, and cell survival 213.

Furthermore, the expression of UBE2C is closely linked to the angiogenic phenotype. Knockdown of UBE2C downregulates key pro-angiogenic factors such as VEGF and MMP-9. Its positive correlation with microvessel density (MVD) and vasculogenic mimicry (VM) has been established in both non-small cell lung cancer and breast cancer, supporting its role in tumor-associated angiogenesis 80, 200, 214.

Tumor necrosis factor receptor 1 (TNFR-1) also represents a key regulator of angiogenic activity. In the mechanism governing angiogenesis regulation, UBE2N specifically catalyzes K63-linked polyubiquitination—a modification distinct from the canonical K48-linked ubiquitination that typically targets proteins for degradation 215. Within this mechanism, UBE2N forms an immune complex with the E3 ligase RNF213, termed the RNF213-UBE2N complex. RNF213 may mediate K63-linked polyubiquitination of TNFR-1 ligands through this complex 216.

In summary, these studies demonstrate that specific E2s critically regulate angiogenesis through distinct mechanisms, such as modulating the stability of key receptors, the expression of angiogenic factors, and the dynamics of specific ubiquitin chain linkages.

### Role of E2s in cellular energy metabolism

Tumor cells exhibit energy metabolism patterns markedly different from normal cells, a characteristic known as metabolic reprogramming, which is a core hallmark of their malignant phenotype 217. Even under adequate oxygen supply, tumor cells preferentially utilize the glycolytic pathway for energy production, a phenomenon termed the Warburg effect. This allows for the rapid generation of ATP and lactate, providing the metabolic foundation for their sustained proliferation and invasion.

E2s play multi-layered regulatory roles in this metabolic rewiring. UBE2C can interact with the epidermal growth factor receptor (EGFR) and enhance its stability, leading to sustained activation of the downstream PI3K-AKT signaling axis. This UBE2C-EGFR-PI3K/AKT regulatory network serves as a critical hub driving metabolic reprogramming and can significantly promote glycolytic flux 130. The activated PI3K-AKT pathway further reinforces the Warburg effect, accelerating the conversion of glucose to lactate and the rapid production of ATP, thereby supplying the necessary bioenergy and biosynthetic precursors for tumor cell proliferation 218.

Beyond the aforementioned pathway, UBE2S can directly catalyze K11-linked polyubiquitination of the VHL protein at lysine residues 171 and 196 in an E3-independent manner, thereby promoting its proteasomal degradation. The loss of VHL indirectly stabilizes the hypoxia-inducible factor HIF-1α, ultimately upregulating the expression of glycolysis-related genes in an HIF-1α-dependent manner 138.

Other E2 members also participate in metabolic remodeling through distinct mechanisms. For instance, the circPDK1/BIN1/UBE2O ternary complex reduces BIN1 stability via ubiquitination, thereby enhancing c-Myc transcriptional activity and driving glycolysis in pancreatic cancer 135. Meanwhile, UBE2Q2 binds to cIAP1 and mediates K63-linked ubiquitination of receptor-interacting protein 1 (RIP1), activating the NF-κB pathway, which upregulates HIF1α transcription and enhances glycolysis 137.

Furthermore, tumor cells can utilize alternative carbon sources such as fatty acids and amino acids for energy supply and biosynthesis, with E2s also playing regulatory roles in these adaptive metabolic processes. This metabolic plasticity enables tumor cells to maintain a survival advantage in diverse microenvironments, thereby acquiring greater potential for progression and treatment resistance.

### Role of E2s in invasion and metastasis

Cancer invasion and metastasis are one of the most critical steps in cancer development and one of the main causes of treatment failure and death. During tumor progression, cancer cells can break through the boundaries of the original tumor, invade surrounding tissues and enter the bloodstream or lymphatic system, eventually forming metastases in other parts of the body 219. This process involves the regulation of complex signaling pathways and molecular mechanisms, among which E2s, as one of the key regulators, play an important role in cancer invasion and metastasis.

Vimentin, a type III intermediate filament protein, serves as a key regulator of EMT, a process critical for tumor invasion and metastasis. UBE2C drives the invasion and metastasis of follicular thyroid carcinoma by mediating K29 site-specific ubiquitination of vimentin, thereby regulating the EMT machinery 220. In addition, a conserved miRNA, miR-525-5p, was found to target UBE2C and subsequently regulate the expression of ZEB1/2 221. Mechanistically, supplementation of UBE2C partially rescues the inhibitory effect of miR-525-5p on cellular invasion. UBE2D1 primarily exerts its pro-metastatic function via the TGF-β/SMAD4 pathway. Knockdown of UBE2D1 reduces the ubiquitination level of SMAD4, thereby attenuating TGF-β/SMAD4 signaling. This leads to downregulation of EMT markers and consequently inhibits cell migration and metastasis in both *in vitro* and *in vivo* models 222.

There are also a number of E2s, such as UBE2T and UBE2J1, that can affect cellular function and thus cancer cell invasion and metastasis through different mechanisms. For example, overexpression of UBE2T may promote prostate cancer cell proliferation and metastasis by activating AKT/GSK3β/β-linker pathway 223. Functionally, UBE2J1 could inhibit the proliferation and metastasis of CRC cells *in vitro* and *in vivo*. The UBE2J1-TRIM25 complex induces ubiquitination and degradation of RPS3 at the K214 residue. Downregulation of RPS3 inhibits NF-κB translocation into the nucleus and therefore inactivates the NF-κB signaling pathway 65.

Based on the integrated analysis of studies in this section, it can be concluded that E2s are not only involved in conventional intracellular protein synthesis and degradation pathways, but also extensively participate in multiple biological processes of tumor cells—including growth, migration, invasion, death, metabolism, and immune microenvironment remodeling—through positive or negative regulation of key signaling pathways and cytokines.

## E2s in enabling characteristics of cancer

### E2s in tumor-promoting inflammatory signaling

In recent years, inflammation has been increasingly recognized as a major hallmark of cancer 224. Aberrant signaling cascades during inflammation promote chronic inflammation, contributing to tumorigenesis 225. E2s play a critical role in regulating inflammatory signaling pathways, and the loss or overexpression of E2s may lead to aberrant inflammatory signaling in specific contexts.

UBE2N influences cancer-promoting inflammatory signals by regulating the TRAF6-mediated NF-κB signaling pathway, a signaling cascade frequently hijacked in tumor-promoting inflammation 226. The NF-κB signaling pathway plays diverse roles in immunity and development and is frequently hijacked to regulate tumor-promoting inflammation. In TLR and RANK signaling pathways, the E3 ligase TRAF6 forms a functional complex with its dedicated E2 partner, UBE2N 227. This TRAF6-UBE2N complex specifically catalyzes K63-linked polyubiquitination, which is required for the subsequent activation of the IKK complex. IKK activation then drives NF-κB signaling, leading to nuclear translocation and pro-inflammatory gene expression 228. Additionally, similar to NF-κB, STAT3 acts as a transcription factor regulating anti-inflammatory responses, and it can limit RANK - and TLR4-mediated signaling by suppressing expression of UBE2N 229. Studies have also found that loss of UBE2N in keratinocytes leads to skin inflammation, increased epithelial cell proliferation, and significant infiltration of immune cells, particularly neutrophils and macrophages. This inflammatory response can be alleviated by affecting the TLR/IL-1 signaling pathway-mediated molecules IRAK1/4 230. Therefore, UBE2N may also participate in promoting cancer-related inflammatory responses by modulating the TLR/IL-1 signaling pathway.

Other E2s associated with inflammatory diseases is UBE2D2, which plays an important role in the inflammatory process of atherosclerosis. Under oxidized ox-LDL stimulation, intracellular expression of miR-30b-5p is upregulated. miR-30b-5p binds to UBE2D2, reducing its ubiquitin conjugation ability, leading to increased stability of KAT2B. The upregulation of KAT2B promotes the acetylation of HMGB1, resulting in the dissociation of HMGB1 from the deacetylase SIRT1, ultimately leading to the release of HMGB1 231, 232. The release of HMGB1 further triggers the inflammatory response and promotes polarization and migration of macrophages 232. Therefore, UBE2D2 participates in the regulation of the inflammatory response by modulating the ubiquitination and acetylation levels of KAT2B and HMGB1 during inflammation. Given the similarity between the inflammatory signals of tumors and the inflammatory process of atherosclerosis, it is speculated that UBE2D2 may play a role in certain types of tumors.

E2s convert chronic, uncontrolled inflammatory responses into drivers supporting malignant tumor progression through precise ubiquitination codes. Future research should no longer view E2s in isolation within a single pathway, but rather focus on their networked functions throughout the entire tumor microenvironment system. By deciphering this intricate network, we may uncover the “master switch” controlling tumor-promoting inflammation, paving the way for novel therapies that reprogram the tumor microenvironment and fundamentally reshape the landscape of cancer treatment.

### E2s in genomic instability and mutation

Genomic instability typically refers to the presence of various DNA alterations, ranging from single nucleotide changes (such as base substitutions, deletions, and insertions) to chromosomal rearrangements (gains or losses of chromosomal segments or entire chromosomes 233. Loss of genomic stability can lead to the early onset or accelerated progression of degenerative diseases, including premature aging and cancer. DNA serves as the template for fundamental processes like replication and transcription; therefore, maintaining its integrity is crucial for cell survival. DNA repair is essential for maintaining genomic integrity for cell survival, and failure to repair DNA damage can lead to genomic instability, cancer, or even cell death 234. The loss or mutation of DNA repair functions is a fundamental cause of genomic instability in hereditary cancers 235. Genomic instability is closely associated with the ubiquitination process, in which E2s such as RAD6, UBE2D3, UBE2I, UBE2L3, and UBE2N play crucial roles.

For example, RAD6 regulates its function in DNA repair by modifying proliferating cell nuclear antigen (PCNA), a key coordinator of DNA replication and repair processes, which participates in various DNA repair pathways through ubiquitination and SUMOylation 236. Besides, RAD6 plays a key role in repairing UV-induced DNA damage, RAD6 physically interacts with HP1α and ubiquitinates HP1α at residue K154, thereby promoting HP1α degradation through the autophagy pathway and ultimately leading to an open chromatin structure that promotes efficient HR DSB repair. In addition, bioinformatics studies have shown that the expression of RAD6 and HP1α has an inverse relationship and is related to the survival rate of patients 237, 238 (Figure 3C). Multiple E2s are critically involved in maintaining genomic integrity through distinct DNA damage response and repair pathway. For instance, downregulation of UBE2D3 enhances the DNA damage repair pathway, leading to increased expression of key proteins such as ATM, ATR, p-ATM, cyclin D1, and CHK1. Concurrently, it results in the suppression of DNA damage and cell cycle regulators, including γH2AX, CDC25A, and CDC25C 169. ZHIYUAN SHEN et al. found that UBE2I interacts specifically with DNA repair-related proteins such as RAD52, RAD51, p53, and UBL1 in a yeast two-hybrid system 6. This specific interaction may affect DNA repair pathways, thereby influencing genomic stability. UBE2L3 regulates the role of 53BP1 in the repair of DSBs by promoting the ubiquitination and degradation of 53BP1 239, a key factor in DNA DSBs By regulating 53BP1 abundance, UBE2L3 modulates the cellular response to replication stress, thereby affecting the choice of DSB repair pathways, which may affect the cell's DNA repair capacity and genomic stability. UBE2N redistributes to the nucleus upon DNA damage and facilitates the assembly of complexes within the RAD6 pathway of DNA repair, preventing genomic instability and cancer. When cells are subjected to DNA damage, UBE2N forms heterodimers with non-catalytic E2-like partner proteins Mms2 and Uev1A, which play a role in the RAD6 pathway of DNA repair 240, similar to UBE2S and UBE2W. UBE2S binds to Ku70 and affects non-homologous end-joining (NHEJ)-mediated DNA repair processes through this interaction, which may affect the accuracy and efficiency of DNA repair 241. UBE2W interacts with DNA repair-related proteins such as BRCA1 and BRCA2, which are involved in the repair of DNA double-strand breaks 49.

Chromosomal instability, which arises from sustained errors in chromosome segregation during mitosis, is the most common form of genomic instability 242. Excessive induction of random chromosomal instability can trigger detrimental chromosomal patterns in cells 243, which may promote tumorigenesis. This process is also regulated by ubiquitination process. UBE2C participates in the activation of the APC/C, an important regulator of cell mitosis 169. Overexpression of UBE2C may induce premature activation of APC/C, resulting in erroneous chromosome segregation and mitotic progression, which directly promotes chromosomal instability. Similarly, downregulation of UBE2D3 leads to increased telomerase activity and upregulation of various proteins in the hTERT and shelterin complexes, such as TRF1, TRF2, POT1, and RAP1. These proteins play a crucial role in protecting telomeres and maintaining chromosomal stability. Thus precise regulation of distinct E2s is critical for maintaining chromosomal stability through multiple mechanisms.

## Targeting E2s to treat cancer

**Figure 5:**
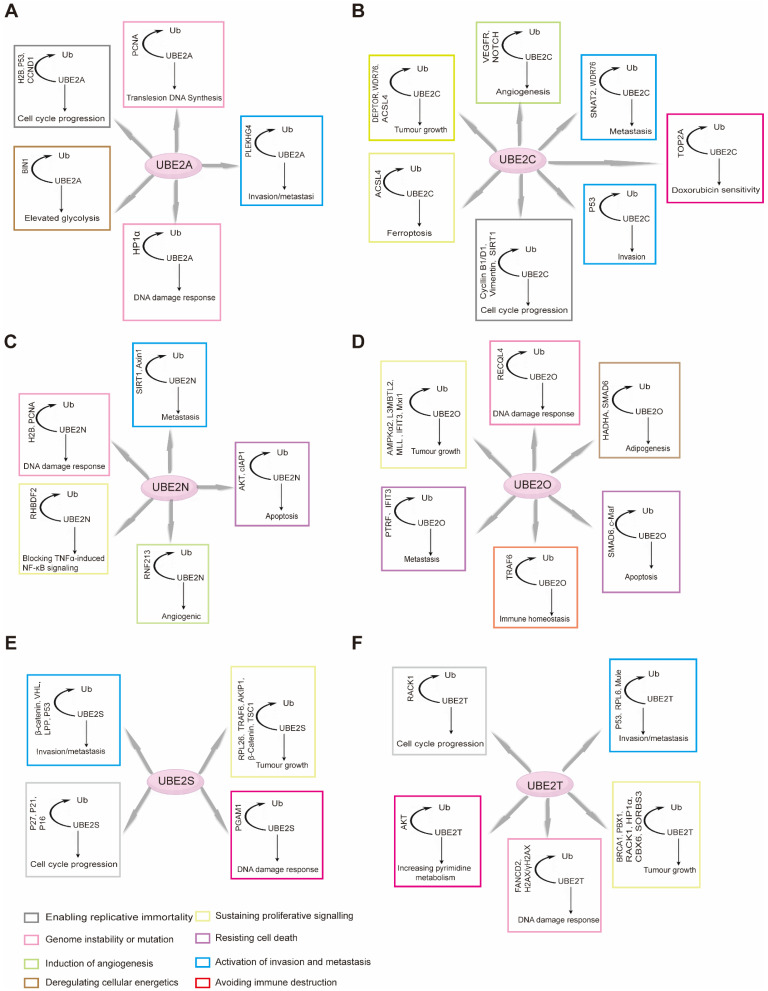
E2 targets in cancer. Ubiquitin-conjugating enzymes (E2s) play multifaceted roles in cancer initiation and progression, with their functions highly dependent on the cellular environment. Most E2s exhibit dual tumor-suppressor or oncogenic activities under specific conditions. Although understanding of E2 mechanisms in tumors remains nascent, several members have emerged as key preclinical targets due to their unique properties and capacity to drive tumorigenesis by regulating multiple cancer hallmarks.

E2 occupies a pivotal position in the ubiquitin-proteasome system, directly determining the fate of substrate proteins and serving as a key regulator of cell cycle progression 53, DNA repair, and signal transduction 244. Therapeutic strategies targeting E2 aim to precisely intercept multiple oncogenic pathways upstream by specifically inhibiting E2 members abnormally activated in cancer, offering a novel approach for achieving highly effective and low-toxicity tumor treatment. E2s in cancer are context-dependent, arising from the specific pathways they regulate. E2s are involved in a variety of physiological functions within cells, and their impact on cancer is often complex. For instance, specific E2 and the ubiquitin ligase Parkin protein mediate the mitophagy pathway in cells, while E2 containing the baculovirus IAP repeat sequence may inhibit the expression of autophagic flux 245. Many E2s exert multiple, sometimes opposing, roles in different cellular environments and pathways (Figure 5). So, is there a perfect target in E2 that deserves to be the focus of cancer drug discovery efforts? This question is not easy to answer. The current literature predominantly focuses on a subset of E2s that are most relevant to cancer and often touted as valuable therapeutic targets. These tend to be well-studied, widely expressed enzymes for which research tools are readily available. Consequently, this focus may have obscured the potential roles of low-abundance, specialized E2s that are dysregulated in specific cancer contexts. Overall, the ratio of charged to uncharged E2 and the concentration of free ubiquitin determine whether the OTU deubiquitinase, ubiquitin aldehyde-binding 1-E2 complex can act as a deubiquitinase or polyubiquitination inhibitor 246. The ubiquitin system plays a pivotal and multifaceted role, representing a rich source of clinical therapeutic targets. Preclinical evidence demonstrates the feasibility of using small molecule inhibitors of E2s to attenuate the degradation of many tumor suppressor substrates (Table 2), inhibit cell proliferation *in vivo*, and inhibit tumor growth *in vivo*.

Structure determines function. To develop E2-targeting inhibitors, it is essential to first understand the structural characteristics of E2s. The UBC domain is the core catalytic unit of E2, responsible for generating the E2-Ub conjugate. This domain typically adopts an α/β-fold structure, consisting of four α-helices and four β-strands, with key loop regions constituting part of the E3-binding site (L1 and L2 loops) and the active site (L3 loop) 11. The highly variable, flexible L1 and L2 loops, extending from the C-terminus to strand 1 and strand 3, are responsible for binding specific E3 ligase 247. The L1 loop in E2 is crucial for this interaction: its sixth residue acts as an interfacial hotspot and is indispensable for E2-E3 interaction. Furthermore, residues at other positions on the L1 loop participate in regulating the specificity of E2-E3 interactions. For example, HECT-type E3 ligases can contact the second residue of the L1 loop, while RING-finger-type E3 ligases do not exhibit this interaction pattern 248. A highly conserved loop anchors H2 to strand 4 and, together with H3, forms a shallow residue groove that accommodates the active site cysteine 247. Structural elements surrounding the active site Cys are involved in processing and activating Ub/Ubl 11, 249. The catalytic groove also houses other conserved residues, the most important of which is the three-residue HPN motif located upstream of the active site cysteine residue. The secondary structural elements of these enzymatic catalytic domains are highly conserved, suggesting the existence of a universal mechanism promoting enzyme function 250.

Current research on the therapeutic potential of targeting E2s remains incomplete, with more comprehensive studies available only for specific E2s such as UBE2T, UBE2C, and UBE2N. UBE2T possesses a typical UBC domain along with an extended, less conserved, and predominantly disordered C-terminal region 250. Structural analysis reveals that its surface lacks obvious deep pockets or stable small-molecule binding sites, which may be one reason for the low success rate in drug development targeting UBE2T. The catalytic activity of UBE2T depends on cysteine 86 (Cys86) 251. Mutation of this residue (C86) completely blocks its ubiquitin transfer function. Additionally, mutation of key lysine sites on its downstream target proteins (K119/120 of H2AX 158, K8/14 of AKT 252) similarly hinders the completion of ubiquitination.

Functionally, UBE2T can cooperate with up to 15 different E3s 253, including Mule 254, INF8 158, FANCL 255, among others. Besides the conserved UBC domain, UBE2T also contains multiple flexible regions that may undergo conformational changes when binding to different E3s; its N-terminal helix exhibits significant conformational rearrangement, swinging from a position distant from the active site to a position closer to FANCL, forming a tighter, more specific binding interface 256. Furthermore, UBE2T itself is subject to ubiquitin modification, containing multiple lysine sites (including Lys28, 48, 91, and 192, etc.); this self-ubiquitination affects protein degradation by regulating UBE2T stability 251. UBE2C possesses a typical UBC domain along with an N-terminal extension of 28 residues. The active site cysteine responsible for catalyzing ubiquitin-adduct formation, Cys114, is located between β4 and a 3_10_ helix. The three-residue-per-turn structure of the 3_10_ helix positions its positively charged Lys119 residue adjacent to the active site Cys114, together facilitating ubiquitin-adduct formation 257. Four β-turns (β1-β4) provide a contact surface for the active site Cys114 258. Additionally, the ubiquitination and degradation of UBE2C itself depend on Cys114 and a D-box-like motif located at residues 129-132 257, 259. UBE2C works closely with the E3 ligase APC/C, together forming a key molecular machinery regulating cell mitosis progression and exit 260. Notably, while the N-terminal extension of UBE2C is not essential for ubiquitin-adduct formation, it participates in regulating APC/C activity as part of an inhibitory mechanism 257. Furthermore, the QNP motif (Gln4, Asn5, and Pro8) within its N-terminal extension exerts a negative regulatory effect on APC/C activity through interaction with APC/C 261. In drug development targeting UBE2C, one of the main challenges stems from the three-dimensional structural features of E2s-their catalytic sites are located in relatively shallow and conserved regions, making it difficult for small-molecule inhibitors to achieve high-affinity, specific binding 262. UBE2N has Cys87 as its catalytic active center 263. The morphology of the active site near this residue is regulated by conformational changes in a flexible loop composed of amino acids 114-124. This loop can adopt different conformations, thereby altering the volume and shape of the active site cavity and significantly affecting the morphology and distribution of potentially druggable binding pockets around Cys87 264. In co-crystal structures with covalent inhibitors, three druggable pockets have been identified in UBE2N 265. The position, shape, and size of these pockets vary with the arrangement of surface amino acids. In UBE2N, the transition of its active site loop from an inactive to an active state may be partially triggered by Asn123 - a residue unique to UBE2N. In the inactive loop conformation, Asn123 is buried within the protein, forming a hydrogen bond network with the backbone carbonyl oxygens of His77, Pro78, and Val80. In the active conformation, Asn123 rotates to the surface, forming a hydrogen bond with the backbone amide of the attacking acceptor ubiquitin's Lys63, thereby driving the structural rearrangement of the entire loop 266, 267. UBE2N interacts with various proteins, including not only E3s but also essential E2 cofactors-the nuclear UBE2V2 and the cytoplasmic UBE2V1. Notably, the binding interfaces between UBE2N and these two cofactors are identical. UBE2V2 and UBE2V1 are highly similar in structure and sequence. Their three-dimensional structures are highly conserved, with the few non-conserved residues located outside the binding interface with UBE2N. Therefore, inhibitors targeting the interaction between UBE2N and UBE2V2 are likely to also affect the binding between UBE2N and UBE2V1 268, 269.

Based on our reading and analysis of the relevant literature, a handful of E2s stand out for their broad and well-established roles in promoting multi-pathway cancer. Many other E2s commonly cited as cancer targets may have both tumor-promoting and tumor-suppressive effects, depending on the environment, providing a higher standard for generating safe small molecule drugs.

Currently reported E2 inhibitors primarily function through three modes of action: binding to the active site, allosteric sites, or protein-protein interaction interfaces 270. Among these, active site inhibitors can be further categorized into covalent and non-covalent types: covalent inhibitors exert their effects by forming irreversible or reversible covalent bonds with the catalytic cysteine residue, whereas non-covalent inhibitors reversibly bind to the target via hydrogen bonds, hydrophobic interactions, van der Waals forces, etc. Covalent inhibitors offer advantages such as prolonged residence time and high binding affinity, which help reduce the risk of drug resistance 271 and maintain high target occupancy even at low plasma concentrations 272. However, selectivity remains a major challenge: high reactivity may lead to off-target modification of non-target proteins, thereby causing off-target toxicity 273. For example, the toxicity of the UBE2N inhibitor BAY 11-7082 in multiple myeloma cells has been shown to be independent of its inhibition of the NF-κB pathway, suggesting off-target effects 274. In contrast, research on non-covalent inhibitors targeting E2s is relatively limited. Although some molecules exhibit good activity *in vitro*, their pharmacokinetic properties often do not align with traditional drug design principles. For instance, non-covalent inhibitors of UBE2N such as Peptoids 275 and ML307 276 failed to advance further in development due to poor metabolic stability or unfavorable overall pharmacokinetic profiles. Future efforts should focus on structural optimization of existing molecular scaffolds to improve key pharmacokinetic parameters such as metabolic stability while maintaining activity. Allosteric inhibitors function by binding to allosteric pockets distinct from the catalytic site, typically offering better selectivity and lower toxicity. However, allosteric sites are often concealed and difficult to identify. Despite the generally shallow active sites of E2s, which lack typical druggable features, successful cases such as the allosteric inhibitor CC0651 277 for UBE2R1 have been reported. This molecule inhibits catalytic function by inducing conformational changes in the enzyme and distorting its active center. This strategy provides an important reference for developing highly selective inhibitors targeting other E2s. Protein-protein interaction (PPI) inhibitors theoretically offer exceptionally high specificity 278. However, PPI interfaces are usually large, flat, and exhibit strong binding affinity, making them difficult to effectively block with small molecules. Candidate molecules in this field often face challenges such as poor solubility and high molecular weight. Currently, research in this area remains in its early stages, but preliminary breakthroughs have been made. For example, C25-140 can effectively inhibit the E3 ligase activity of TRAF6 by specifically disrupting the interaction between TRAF6 and UBE2N 279.

In addition to using small molecule inhibitors to directly target the structure of E2s, using miRNAs to inhibit E2 mRNAs may also affect the protein turnover process of cancer cells. MiRNA is an endogenous small non-coding RNA involved in the regulation of gene expression (Table 3) 280. These molecules potently suppress the expression of their target genes, thereby inhibiting cell growth *in vitro* and tumor growth *in vivo*. Compared with plasmid DNA-based gene therapy and protein-based drug molecules, miRNA, as a natural antisense nucleotide, exhibits lower immune response and low toxicity 281. Although miRNA drugs can target molecules that cannot be targeted by chemical drugs or antibody drugs, it is difficult to achieve ideal pharmacological effects due to the low stability of miRNA and its difficulty in delivering into cells.

Currently, among specific components in UPP, inhibitors targeting E3s and DUBs have advanced to clinical trials for treat cancer, and inhibitors targeting E2 are still in the basic research stage 282. Nevertheless, E2s serve as promising therapeutic targets, and their potential should not be overlooked. Therefore, further investigations need to be carried out to evaluate the feasibility and sensitivity of E2 as a potential target. E2-targeted-strategies need to be more effective, sensitive and safer as a therapeutic target for cancer.

## Conclusions and Perspective

Over the past decade, our understanding of E2s within the ubiquitin-proteasome system and their roles in tumor biology has significantly deepened. While targeting E2s has emerged as a promising therapeutic strategy in oncology, their clinical translation faces critical challenges. These primarily stem from the functional complexity of E2s, their inherent "undruggability" due to suboptimal binding site characteristics, and the potential toxicity risks associated with systemic inhibition.

This review systematically elucidates that E2s, by dynamically modifying a diverse array of substrates, are integral to core processes driving tumorigenesis and progression. Notably, certain E2s, such as UBE2D3 and UBE2L3, exhibit a "dual-role" functionality in cancer. Their biological impact is highly contingent on the specific E3 ligase they cooperate with and the particular substrate they modify. For instance, UBE2D3, when paired with MDM2, catalyzes K48-linked polyubiquitination of the tumor suppressor p53, leading to its proteasomal degradation—a key mechanism enabling cancer cells to evade growth suppression and apoptosis (Figure 4C) 313. Conversely, in concert with KLHL13, UBE2D3 mediates K63-linked polyubiquitination of TAP2 at lysine 245, thereby impairing antigen presentation pathways and anti-tumor immunity in pancreatic cancer 141. Similarly, UBE2L3 can promote the ubiquitination and degradation of 53BP1 314, suppressing high-fidelity homologous recombination repair and exacerbating genomic instability, while it also facilitates the degradation of GSK-3β, promoting EMT and metastasis 315. This context-dependent duality underscores the central, yet complex, position of E2s in oncogenic signaling networks and highlights the necessity for highly precise and selective targeting strategies in future therapeutic development.

Looking ahead, overcoming the current bottlenecks in E2-targeted therapy requires a concerted effort across three dimensions. First, at the level of basic research, a deeper mechanistic dissection is imperative. Future studies must systematically delineate the functional networks, regulatory logic, and downstream signaling crosstalk of individual E2s within specific biological contexts and tumor microenvironments. This foundational knowledge is crucial for developing selective intervention strategies, such as those targeting specific E2-E3 pairs, distinct ubiquitin chain linkages, or particular substrate modification events.

Second, in drug discovery, innovative pharmacological paradigms are needed to overcome the "undruggable" nature of many E2s. The typically shallow and flat active sites of E2s pose a significant hurdle for conventional small-molecule inhibitors. Future directions should focus on: 1) developing novel chemotypes with high potency through structure-based rational design or mining of natural products; 2) embracing protein degradation technologies, such as PROTACs and molecular glues, to shift the paradigm from "functional inhibition" to "protein elimination"; and 3) exploiting covalent targeting strategies for E2s with suitable residues to enhance binding affinity and selectivity.

Third, regarding therapeutic strategy, optimized approaches are essential to balance efficacy and safety. Given the global role of E2s in maintaining proteostasis, broad inhibition may incur significant toxicity. Smarter strategies include: 1) exploring the E2-inhibitory activities of already approved drugs (e.g., ATO 316) to expedite clinical translation, and 2) repositioning E2 inhibitors as "sensitizers" or combination partners with chemotherapy, radiotherapy, or other targeted agents. Such synergistic approaches could lower effective doses, improve therapeutic indices, and overcome resistance.

The emergence of novel therapeutic modalities is fundamentally expanding the design landscape for E2-targeting drugs. Among these, proteolysis-targeting chimeras (PROTACs 317-318) and molecular glue technologies 319 represent a paradigm shift—from traditional "occupancy-driven inhibition" to "event-driven clearance." As bifunctional molecules, PROTACs simultaneously recruit the target protein and an E3 ligase, inducing ubiquitination and subsequent proteasomal degradation of the target. Molecular glues, in a more refined manner, induce or stabilize interactions between the target protein and an E3 ligase or other degradation machinery components, thereby "hijacking" the target into the degradation pathway. The core advantage of both strategies lies in their catalytic mechanism of action: instead of relying on prolonged target occupancy, they operate by triggering discrete degradation events. Consequently, these approaches often demonstrate high potency at low doses and hold promise for overcoming resistance caused by target overexpression or mutation.

In conclusion, E2s represent a promising frontier in cancer therapy, yet their path to clinical application is fraught with mechanistic complexity and technical hurdles. Future breakthroughs will depend on the synergistic integration of three pillars: deepening mechanistic insights, diversifying drug development technologies, and intelligent clinical strategizing. Through the convergence and collaborative innovation of structural biology, chemical biology, computational science, and clinical oncology, we can anticipate the eventual translation of E2-targeted therapies into precise and effective treatments for cancer patients.

## Figures and Tables

**Table 1:** E2s in the cancer hallmarks

Cancer hallmark	E2s	Cancer hallmark	E2s
Sustained proliferative signaling 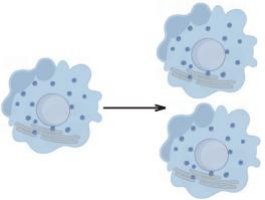	UBE2A 53, UBE2B 54, UBE2C 55, 56, UBE2D3 57, UBE2E1 58, 59, UBE2E2 60, UBE2E3 61, UBE2F 62, UBE2I 63 UBE2J1 64, 65, UBE2L3 66, UBE2M 67, UBE2O 68, 69, UBE2Q 1^70^ UBE2Q2 71, UBE2R1 72, UBE2T 73, 74, UBE2V1 75, UBE2V2 76, 77, UBE2Z^78^.	Inducing angiogenesis 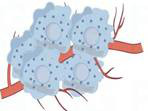	UBE2C 79, 80, UBE2D1 81, UBE2D3 82, UBE2E1 83, UBE2L6 83, UBE2O 84.
Enabling replicative immortality 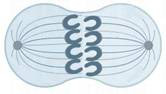	UBE2D3 85, 86, UBE2N 87, 88, UBE2T 89.	Tumor-promoting inflammation 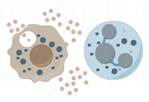	UBE2C 90, 91, UBE2I 92, UBE2L3 93, UBE2L6 94, UBE2N 95.
Evading growth suppressors 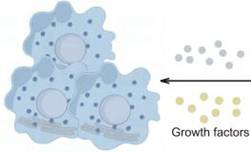	UBE2C 55, UBE2D1 96, UBE2E3 97, UBE2F 98, UBE2I 99, UBE2L3 100, UBE2M 101, UBE2N 102, UBE2Q1 103, UBE2R1 104, UBE2S [105]UBE2T 106.	Inducing invasion and metastasis 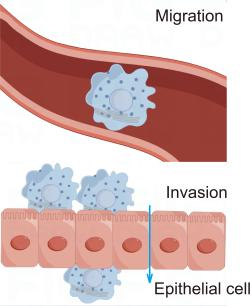	UBE2A 107, UBE2B 108, UBE2C 109, 110, UBE2D3 111, UBE2E2 112, UBE2F 113, UBE2I 114, UBE2J1 65, UBE2O 115, UbE2T 73, 74, 116, UBE2V1 75.
Resisting cell death 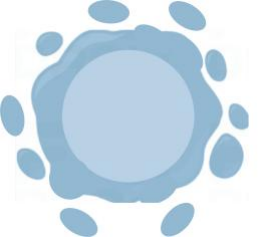	UBE2A 117, UBE2C 56, 118, UBE2D1 80, 119, UBE2D2 120, UBE2D3 121, UBE2E1 58, UBE2E3 61, UBE2F 122, UBE2J1 64, UBE2L3 123, 124, UBE2L6 125, UBE2M 67, UBE2O 126, UBE2S 127, UBE2T 116, 128, UBE2V2 76, 77.	Deregulating cellular energetics 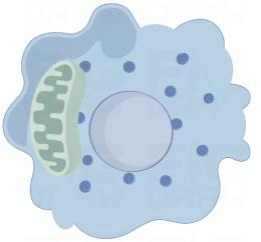	UBE2A 129, UBE2C 130-132, UBE2F 62, UBE2I 133, UBE2N 134, UBE2O 135, UBE2Q1 136, UBE2Q2 137, UBE2S 138, UBE2T 139.
Avoiding immune destruction 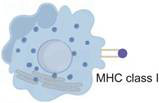	UBE2C 140, UBE2D3 141, UBE2F 113, UBE2I 142, UbE2M 143, UBE2N 144, UBE2R1 145, UBE2S 146, UBE2T 147, UBE2V2 148, UBE2W 49.	Genomic instability and mutation 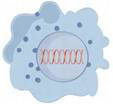	UBE2A 149, UBE2B 149, UBE2C 150-152, UBE2E2 60, UBE2I 153, 154, UBE2L3 93, UBE2N 155, UBE2O 156, UBE2S 157, UBE2T 158.

**Table 2:** Inhibitors targeting E2s

Name	Chemical structure	Molecular formula	Inhibitor category	Origin	Target	Mechanism of action	*In vitro inhibition rate*	Tumor inhibition rate	Cancer Type	Refs
TZ8	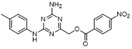	C_18_H_16_N_6_O_4_	Non-covalent inhibitor	Synthesis	UBE2B	Inhibits H2A ubiquitination.	IC_50_:25μM (MDA-MB-231)	-	TNBC	283
TZ9	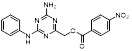	C_17_H_14_N_6_O_4_	Non-covalent inhibitor	Synthesis	UBE2B	Inhibits H2A ubiquitination.	IC_50_: 6μML (MDA-MB-231)	-	TNBC	283
New triazines	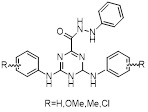	R=H: C_17_H_19_N_7_OR=OMe: C_19_H_23_N_7_O_3_R=Me: C_19_H_23_N_7_OR=Cl: C_17_H_17_Cl_2_N_7_O	Non-covalent inhibitor	Based on TZ8 / TZ9 synthesis	UBE2B	Inhibits H2A ubiquitination.	IC_50_: 3.3 to 22μM (OV90, A2780, H1299, A549, MCF-7, MDA-MB231 and HT29)	-	OC, BC, CRC	284
EN450	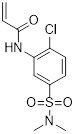	C_11_H_13_ClN_2_O_3_S	Allosteric inhibitor	Synthesis	UBE2D1	Covalently binds UBE2D and exhibits molecular glue interactions with NFKB1.	Approximately 75% inhibition rate at 5μM (HAP1)	-	CML	285
PC3-15	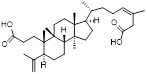	C_32_H_52_O_4_	PPI inhibitor	Natural product compounds	UBE2D2	Binds UBE2D2 and inhibits p62 ubiquitination	EC_50_: 13.5μM (MDA-MB-468)	MDA-MB-468 and HCC1806 xenografts: Lapatinib & PC3-15: 50 mg/kg (approximately 50%)	TNBC	286
DHPO	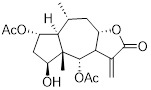	C_10_H_18_O_3_	Non-covalent inhibitor	Natural product compounds	UBE2D3	Binds to UBE2D3 and inhibits NF-κB activation	IC_50_: 2.5 to 8.5μM (CFPAC1, HPAC, BxPC3, Panc1, SW1990, Capan2, AsPC1, and HPNE)	Panc1 in situ tumor model: 10mg/kg (80.67%) SW1990 in situ tumor model: 10mg/kg (78.74%)	PC	222
HA-9104	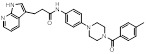	C_28_H_27_N_5_O_2_	Non-covalent inhibitor	Synthesis	UBE2F	Impairs UBE2F-NEDD8 thioester formation and inhibits Cullin-5 neddylation	IC_50_: 5.26μM (H1650) IC_50_: 1.7625μM (H2170) IC_50_: 4.377μM (H358)	H1650 xenograft models: 30 mg/kg (approximately 30%)	LC, PC	287
CW3	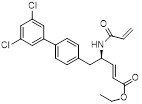	C_22_H_21_Cl_2_NO_3_	PPI inhibitor	Synthesis	UBE2G2	Forms a covalent bond with the thiol group of Cys48 of UBE2G2.	>50% inhibition rate in 24 cell lines (10μM)	-	-	288
2-D08	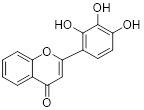	C_15_H_10_O_5_	Non-covalent inhibitor	Synthesis	UBE2I	Induces ROS accumulation-mediated intrinsic apoptosis possibly through deSUMOylation of NOX2.	IC_50_: 18μM (MOLM13) IC_50_: 15μM (ML2)	-	CML	289
NSC697923	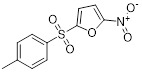	C_11_H_9_NO_5_S	PPI inhibitor	Synthesis	UBE2N	Inhibits UBE2N-Ub conjugate formation, activates p53 and JNK pathways. Inhibits UBE2N-Ub conjugate formation, activates the MEK/FRA1/SOX10 signaling cascade.	IC_50_: 1 to 5μM (IMR32, NGP, NB19, CHLA-255, SK-N-AS, and, SH-SY5Y) IC_50_: 4 to 8μM (A375, A2058, and B16)	SH-SY5Y and NGP orthotopic model: 5 mg/kg (approximately 85%) A375 xenograft model: 5 mg/kg (approximately 75%)	NB, MM	269, 290
UC-764864	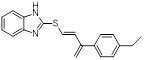	C_19_H_18_N_2_OS	Covalent inhibitor	Synthesis	UBE2N	Interferes with UBE2N to eliminate the function of hematopoietic stem/progenitor cells in leukemia.	IC_50_:4.3 to 6.7 µM (HL-60, NOMO-1, Kasumi-1, MV4-11, and MDSL)	-	AML	263
ATO	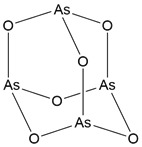	As_2_O_3_	Covalent inhibitor	Natural product compounds	UBE2O	Inhibits the ubiquitination and degradation of AMPKα2, suppressing the activation of the mTOR-HIF1α pathway.	-	UBE2O^+/+^ orthotopic tumor model: 2.5 mg/kg (approximately 75%)	BC, PC	44
CC0651	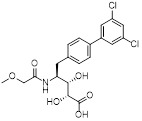	C_20_H_21_Cl_2_NO_6_	Allosteric inhibitor	Synthesis	UBE2R1	Stabilizes UBE2R1-Ub interaction,Impairs Ub transfer.	IC_50_: 1.72 μM (PC-3 and HCT116)	-	PC, CRC	277
CU2	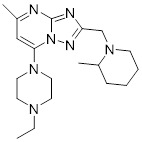	C_19_H_31_N_7_	PPI inhibitor	Synthesis	UBE2T	Blocks UBE2T/FANCL-mediated monoubiquitination of FANCD2, thereby enhancing cellular sensitivity to the DNA-crosslinking agent carboplatin.	-	-	OC	291
M435-1279	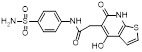	C_18_H_17_N_3_O_5_S_2_	Non-covalent inhibitor	Synthesis	UBE2T	Inhibits the excessive activation of the Wnt/β-catenin signaling pathway by blocking UBE2T-media-ted degradation of RACK1.	IC_50_: approximately 10 μM (HGC27, MKN45, and AGS)	-	GC	292

**Table 3:** miRNAs targeting E2s

Name	Target	IMechanism of action	Cancer Type	Refs
miR-527	UBE2A	Binding to 3′-UTR of UBE2A	HCC	293
miR-455-5p	UBE2B	Binding to 3′-UTR of UBE2B/UBE2V1	OC	294
miR-17/20	UBE2C	Binding to 3′-UTR of UBE2C	GC	295
miR661-3p	UBE2C	Binding to the 3′-UTR of UBE2C	NSCLC	280
miR-381-3p	UBE2C	Directly targeting UBE2C	PC	296
miR-140-3p	UBE2C	Directly targeting UBE2C	OS	297
miR-483-3p	UBE2C	Binding to the 3′-UTR of UBE2C	PC	298
miR-525-5p	UBE2C	Binding to the 3′-UTR of UBE2C	CRC	221
miR-379-5p	UBE2E3	Binding to the 3′-UTR of UBE2E3	BC	61
miR-101	UBE2H, UBE2N	Directly targeting UBE2H/UBE2N	LUAD, BC	299, 300
miR-30a	UBE2H	Directly targeting UBE2H	LUAD	299
miR-30b	UBE2H	Directly targeting UBE2H	LUAD	299
miR-214	UBE2I	Binding to 3′-UTR of UBE2I	GBM	301
miRNA-10a-5p	UBE2I	Binding to 3′-UTR of UBE2I	CRC	302
miR-122-3p	UBE2I	Directly targeting UBE2I	LC	303
miR-147b	UBE2N	Binding to 3′-UTR of UBE2N	HCC	304
miR-590-3p	UBE2N	Directly targeting UBE2N	CRC	305
miR-205	UBE2N	Directly targeting UBE2N	BC	306
miR-31	UBE2N	Directly targeting UBE2N	BC	306
miR-934	UBE2N	Binding to 3′-UTR of UBE2N	BCa	102
miR-671-5p	UBE2R1	Binding to 3′-UTR of UBE2R1	OS	104
miR-1305	UBE2T	Binding to 3′-UTR of UBE2T	HCC	281
miR-1322	UBE2T	Binding to 3′-UTR of UBE2T	HCC	205
miR-543	UBE2T	Directly targeting UBE2T	BC	307
miR-212-5p	UBE2T	Binding to 3′-UTR of UBE2T	HCC	308
miR-498	UBE2T	Binding to 3′-UTR of UBE2T	MEL	309
MiR-182-5p	UBE2T	Directly targeting UBE2T	RCC	310
miR-490-5p	UBE2T	Binding to 3′-UTR of UBE2T	NSCLC	311
miR-605-5p	UBE2T	Directly targeting UBE2T	HCC	312
miR-548c-3p	UBE2T	Directly targeting UBE2T	HCC	312
miR-4689	UBE2V1	Directly targeting UBE2V1	PC	75
miR-499a	UBE2V2	Binding to 3′-UTR of UBE2V2	PCa	76

## Abbreviations

53BP1: P53-binding protein 1
ABC-DLBCL: activated B-cell-like diffuse large B-cell lymphoma
ACSL4: acyl-CoA synthetase long-chain family member 4
AKIP1: a kinase interacting protein 1
AKT: protein kinase B
AMPKα2: AMP-activated protein kinase catalytic subunit alpha 2
Apaf1: apoptotic protease-activating factor 1
APC/C: anaphase-promoting complex/cyclosome
ATP: adenosine triphosphate
Axin1: axis inhibition protein 1
BAX: BCL-2-associated X protein
BC: breast carcinoma
BCa: bladder cancer
BCL2: B-cell lymphoma 2
Bim: Bcl-2 interacting mediator of cell death
BIN1: bridging integrator 1
BRCA1: breast cancer gene 1
BRCA2: breast cancer gene 2
Caspase-3: cysteinyl aspartate-specific protease-3
Caspase-9: cysteinyl aspartate-specific protease-9
CBX6: chromobox homolog 6
CBL: Casitas B-lineage lymphoma proto-oncogene
CCNB1: cyclin B1
CCND1: cyclin D1
CDC25A: cell division cycle 25 homolog A
CDC34: cell division cycle 34
CDK1: cyclin-dependent kinase 1
CGAS: cyclic GMP-AMP synthase
CK2: casein kinase 2
CLAP1: cellular inhibitor of apoptosis protein 1
C-Maf: v-maf musculoaponeurotic fibrosarcoma oncogene homolog
CML: chronic myeloid leukemia
CNOT4: CCR4-NOT transcription complex subunit 4
CNV: copy number variation
CRC: colorectal cancer
CSF3R: colony stimulating factor 3 receptor
Cue1: coupling of ubiquitin conjugation to ER degradation protein 1
DEPTOR: DEP domain containing mTOR-interacting protein
EC: endometrial cancer
EGFR: epidermal growth factor receptor
ER: endoplasmic reticulum
ERK: extracellular signal-regulated kinase
E1: ubiquitin-activating enzyme
E2: ubiquitin-conjugating enzyme
E3: ubiquitin-protein ligase
FANCD2: fanconi anemia complementation group D2 protein
FAS: first apoptosis signal receptor
FASL: fas ligand
FL: follicular lymphoma
GBC: gallbladder carcinoma
GBM: glioblastoma
GC: gastric cancer
GCB-DLBCL: germinal center B-cell-like diffuse large B-cell lymphoma
H2AX/γH2AX: H2A histone family member X/Ser139 phosphorylated H2AX
H2B: histone H2B
H3K4me3: histone H3 lysine 4 trimethylation
H4K4: histone H4 lysine 4
HADHA: hydroxyacyl-CoA dehydrogenase trifunctional multienzyme complex subunit alpha
HCC: hepatocellular carcinoma
HIF-1α: hypoxia inducible factor 1 subunit alpha
HMGB1: high mobility group box 1
HP1α: heterochromatin protein 1 alpha
HSP90AB1: heat shock protein 90kDa alpha, class B member 1
hTERT: human telomerase reverse transcriptase
IAP: inhibitor of apoptosis protein
ICC: intrahepatic cholangiocarcinoma
IDH1/2: isocitrate dehydrogenase 1 and 2
IFIT3: interferon-induced protein with tetratricopeptide repeats 3
IFN-I: type I interferon
IFN-γ: interferon-gamma
IGFBP3: insulin-like growth factor binding protein 3
IKK: IκB kinase
K9me3: histone H3 lysine 9 trimethylation
KAT2B: K(lysine) acetyltransferase 2B
KLHL13: Kelch-like protein 13
L3MBTL2: lethal(3) malignant brain tumor-like protein 2
LPP: LIM domain containing preferred translocation partner in lipoma
LUSC: lung squamous cell carcinoma
MDM2: mouse double minute 2
Mix1: Mix family homeobox 1
MEL: melanoma
MLL: mixed lineage leukemia
MMP-9: matrix metalloproteinase-9
Mms2: methyl methanesulfonate sensitivity protein 2
mTOR: mammalian target of rapamycin
Mule: ARF-binding protein 1
NF-κB: nuclear factor kappa-light-chain-enhancer of activated B cells
NOTCH: Notch receptor
NOXA: NADPH oxidase activator
Noxa: phorbol-12-myristate-13-acetate-induced protein 1
NSCLC: non-small cell lung cancer
OS: osteosarcoma
OTU: ovarian tumor domain protease
P16: cyclin-dependent kinase inhibitor 2A
P21: cyclin-dependent kinase inhibitor 1A
P27: cyclin-dependent kinase inhibitor 1B
P27Kip1: p27 kinase inhibitory protein 1
P53: tumor protein 53
PBX1: pre-B-cell leukemia homeobox 1
PC: pancreatic cancer
PCa: prostatic carcinoma
PCNA: proliferating cell nuclear antigen
PGAM1: phosphoglycerate mutase 1
PI3K: phosphatidylinositol 3-kinase
PLEKHG4: DEP domain containing mTOR-interacting protein
POT1: protection of telomeres 1
PTRF: polymerase I and transcript release factor
Puma: P53 upregulated modulator of apoptosis
RACK1: receptor for activated C kinase 1
RAD51: radiation sensitive 51
RAD52: radiation sensitive 52
RAD6: radiation sensitive 6
RECQL4: RecQ like helicase 4
RHBDF2: rhomboid 5 homolog 2
RNF19A: ring finger protein 19A
RNF213: ring finger protein 213
RPL26: ribosomal protein L26
RPL6: ribosomal protein L6
SARC: sarcoma
SCFβTRCP: skp1-Cul1-F-box protein (β-transducin repeat-containing protein) complex
SIRT1: sirtuin 1
SMAD4: suppressor of mothers against decapentaplegic homolog 4
SMAD6: SMAD family member 6
SNAT2: sodium-coupled neutral amino acid transporter 2
SNV: single nucleotide variant
SORBS3: sorbin and SH3 domain containing 3
TACE: tumor necrosis factor-α converting enzyme
TAP2: transporter associated with antigen processing 2
TGF-β: transforming growth factor-beta
TNF: tumor necrosis factor
TOP2A: DNA topoisomerase II alpha
TRAF6: TNF receptor-associated factor 6
TRF1: telomeric repeat-binding factor 1
TRF2: telomeric repeat-binding factor 2
TSC1: tuberous sclerosis complex 1
Ubl: ubiquitin-like protein 1
UPP: ubiquitin-proteasome pathway
VEGFR: vascular endothelial growth factor receptor
VEGFR2: vascular endothelial growth factor receptor 2
VEGF-A: vascular endothelial growth factor A
VHL: von Hippel-Lindau tumor suppressor
WDR76: WD repeat domain 76
β-TrCP: β-transducin repeat-containing protein
